# Joint models for longitudinal and time-to-event data: a review of reporting quality with a view to meta-analysis

**DOI:** 10.1186/s12874-016-0272-6

**Published:** 2016-12-05

**Authors:** Maria Sudell, Ruwanthi Kolamunnage-Dona, Catrin Tudur-Smith

**Affiliations:** Department of Biostatistics, Block F Waterhouse Building, University of Liverpool, 1-5 Brownlow Street, Liverpool, L69 3GL UK

**Keywords:** Joint model, Meta-analysis, Longitudinal, Time-to-event, Review, Reporting standards

## Abstract

**Background:**

Joint models for longitudinal and time-to-event data are commonly used to simultaneously analyse correlated data in single study cases. Synthesis of evidence from multiple studies using meta-analysis is a natural next step but its feasibility depends heavily on the standard of reporting of joint models in the medical literature. During this review we aim to assess the current standard of reporting of joint models applied in the literature, and to determine whether current reporting standards would allow or hinder future aggregate data meta-analyses of model results.

**Methods:**

We undertook a literature review of non-methodological studies that involved joint modelling of longitudinal and time-to-event medical data. Study characteristics were extracted and an assessment of whether separate meta-analyses for longitudinal, time-to-event and association parameters were possible was made.

**Results:**

The 65 studies identified used a wide range of joint modelling methods in a selection of software. Identified studies concerned a variety of disease areas. The majority of studies reported adequate information to conduct a meta-analysis (67.7% for longitudinal parameter aggregate data meta-analysis, 69.2% for time-to-event parameter aggregate data meta-analysis, 76.9% for association parameter aggregate data meta-analysis). In some cases model structure was difficult to ascertain from the published reports.

**Conclusions:**

Whilst extraction of sufficient information to permit meta-analyses was possible in a majority of cases, the standard of reporting of joint models should be maintained and improved. Recommendations for future practice include clear statement of model structure, of values of estimated parameters, of software used and of statistical methods applied.

**Electronic supplementary material:**

The online version of this article (doi:10.1186/s12874-016-0272-6) contains supplementary material, which is available to authorized users.

## Background

Joint modelling of longitudinal and time-to-event data is an area of increasing research [1–3], which allows the simultaneous modelling of a longitudinal (repeatedly measured over time) outcome such as weekly biomarker measurements, and a time-to-event (survival) outcome such as time to death. The model consists of two sub-models; a longitudinal sub-model (such as a linear mixed effects model) and a time-to-event sub-model (such as a cox proportional hazards models) which are linked using an association structure that quantifies the relationship between the outcomes of interest.

Within a single study, joint models have the potential to reduce parameter estimate bias, account for dropout in longitudinal studies and enable the inclusion of longitudinal covariates measured with error in time-to-event models [1, 4]. These qualities often make joint models preferable to separate longitudinal or time-to-event analyses. Joint models have been applied in the literature to investigate links between biomarkers and certain disease events (e.g. in cancer studies), and to account for informative study dropout.

Glass 1976 [5] defined meta-analysis (MA) as the statistical analysis or pooling of results from separate studies. Such analyses can increase power and precision compared to original studies, or answer questions additional to those originally posed [6]. MA can be performed on the original Individual Participant Data (IPD), or on the study level results (published in the literature or obtained from authors) termed Aggregate Data (AD). Overviews of MA methodology can be found in Whitehead 2002 [7] and in the Cochrane handbook [6].

Whilst the benefits of joint modelling methods for individual studies are well established, little attention has been given towards the potential value of pooling estimates across similar studies in an aggregate data meta-analysis (AD-MA) of joint models. However before an AD-MA using published data can be undertaken, relevant studies must be identified, and the necessary information must be extracted. We aim to investigate the reporting of joint longitudinal and time-to-event models applied to real medical data in the literature to establish whether current reporting practices would allow sufficient data to be extracted to undertake AD-MA.

## Methods

### Identification of papers

We performed our systematic review in accordance to the guidelines of the Preferred Reporting of Items for Systematic Reviews and Meta-Analyses (PRISMA) [8]. We searched the Medline, Pubmed and Scopus datasets for studies using joint models for longitudinal and time-to-event data to analyse medical data (search strategies available in the Additional file 1).

Papers mentioning joint models for longitudinal (or repeated measures over time) data and time-to-event (or survival, event time or event history) data were identified. Duplicates were identified and removed. Abstracts and keywords were then examined, and irrelevant papers were removed. Examples of disregarded papers include papers modelling body joints, papers discussing joint models as a future extension or alternative to methods used, or papers using two stage approaches rather than simultaneous estimation of the longitudinal and time-to-event sub-models. Papers not relating to medical or biostatistical datasets were discarded (e.g. data from plant or animal subjects except from modelling of human diseases input into animal hosts). Additionally papers involving repeated measures over space rather than time were discarded (e.g. repeated measures across tumour sites). If study relevancy was unclear from the abstract, the full text was obtained and viewed after which the study was included or discarded.

Any retained papers were sorted into an applied and a methodological group. Some methodology papers presented results from application to example datasets. These were considered reanalyses of data or demonstrations of methods rather than primary analyses to influence future practice. Also, methodological papers might be expected to better report results as their authors are experts in the area. The aim of this review was to assess how well joint models are reported in the general medical literature, so we focussed on the applied group only.

### Data extraction

A blank data extraction form is presented in Additional file 2. During the investigation we refer to references identified as applying joint models to relevant datasets as studies. Other publications (e.g. those cited by studies) are referred to as papers. Information recorded from identified studies included publication year, author, journal, joint model type, sharing structure between the longitudinal and time-to-event sub-models, types of sub-models, Bayesian or frequentist methods, and software used. Disease area was recorded (with respect to the type of longitudinal and time-to-event data, for example studies modelling biomarkers in heart disease patients after a transplant operation were classed as transplant data).

The sources of the methods used were recorded. Specifically if the study developed methods specific to their dataset, “own methods developed” was recorded. If the study referenced specific papers as the source of the methods they used the papers referenced were recorded.

Availability of information required for a MA was also recorded (including the number of participants, significance level, and presence longitudinal, time-to-event and association parameters along with their precision estimates). The significance level used was identified through direct statement in the text, or specified confidence interval sizes on tables, graphs, or footnotes relating to the joint models fitted. For a MA to be considered possible, the number of participants and model coefficients had to be reported, with either a standard error, or a confidence interval with accompanying significance level.

We assume for an AD-MA of joint longitudinal and time-to-event models that a separate MA would be conducted for each of the longitudinal, the time-to-event and the association parameters from the identified studies. Consequently when identified studies were assessed for sufficient information to conduct a MA, they were assessed separately for longitudinal, for time-to-event and for association information. Ideally all three separate groups of MA would be conducted, however if insufficient information was reported only a subset of these MA might be feasible.

The reason for joint models use (see Henderson et al [9) may influence what information is presented in the study report. If joint models were used to account for informative dropout, the study might not report the time-to-event parameter information (although if the time-to-event endpoint is clinically defined then time-to-event estimates should be reported). Similarly if interest was to include a longitudinal variable measured with error as a time-variable covariate in a time-to-event model, the study may not clearly report longitudinal sub-model parameters. To investigate whether the reason for joint model use affected the proportion of possible MA for each of the longitudinal, time-to-event and association parameter components, we investigated the proportions of possible MA for studies using joint models to account for dropout, or to account for error in a time-varying covariate.

The aim of this review was not to perform any MA, solely to assess if MA were undertaken, what proportion of the identified studies could contribute.

## Results

**Fig. 1:**
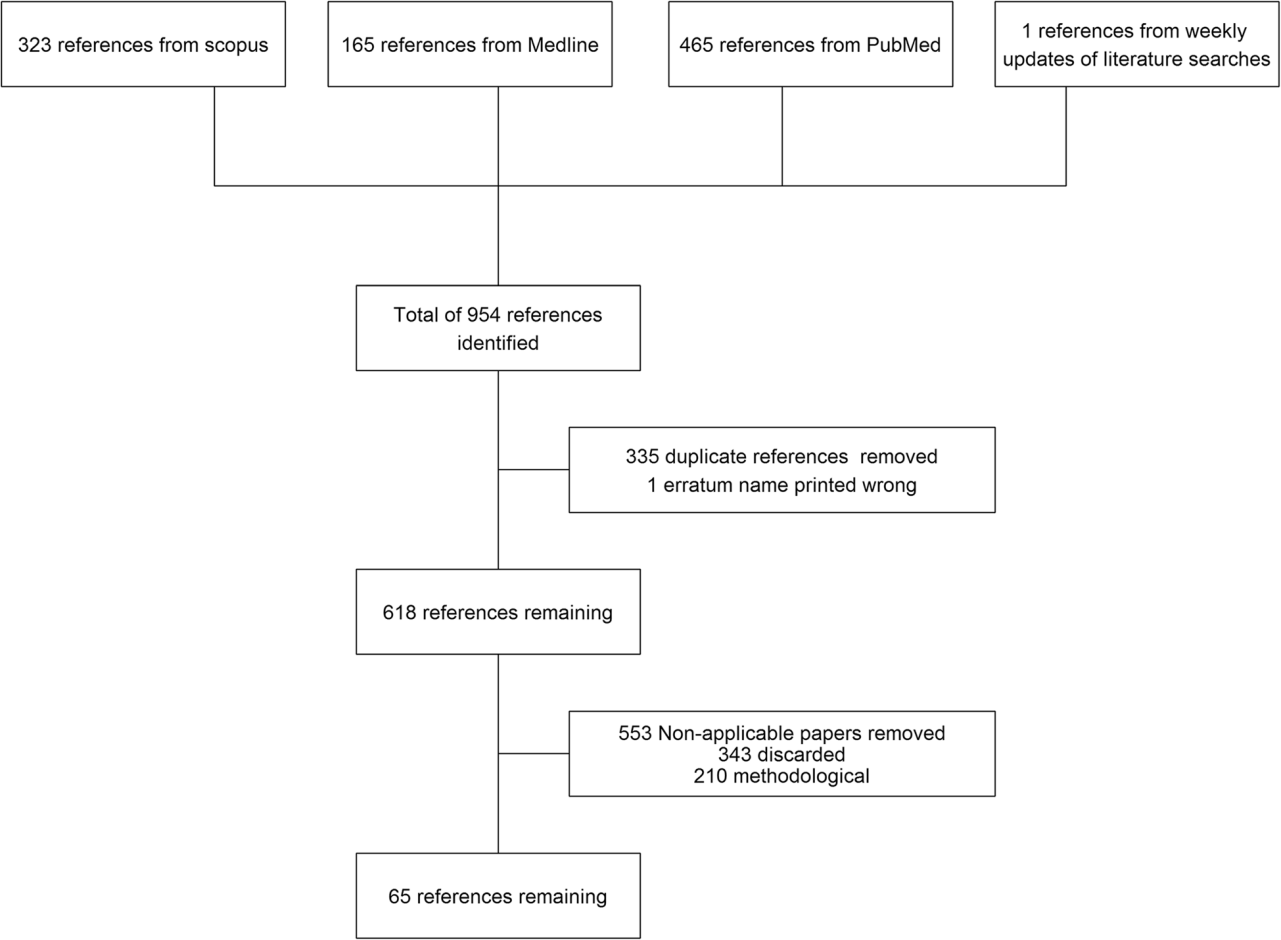
Flowchart of study identification

Searches were conducted on the 15th September 2015. The number of references identified is shown in Fig. 1. Once duplicate references were removed, (and an erratum paper correcting an author’s name), 618 references remained. Of these, we identified 210 methodological papers, and disregarded 343 references. In total 65 studies [9–73] remained that applied joint models to data with the aim of influencing healthcare rather than solely presenting new joint modelling methods.

### Characteristics of identified studies

#### Year of publication

**Fig. 2:**
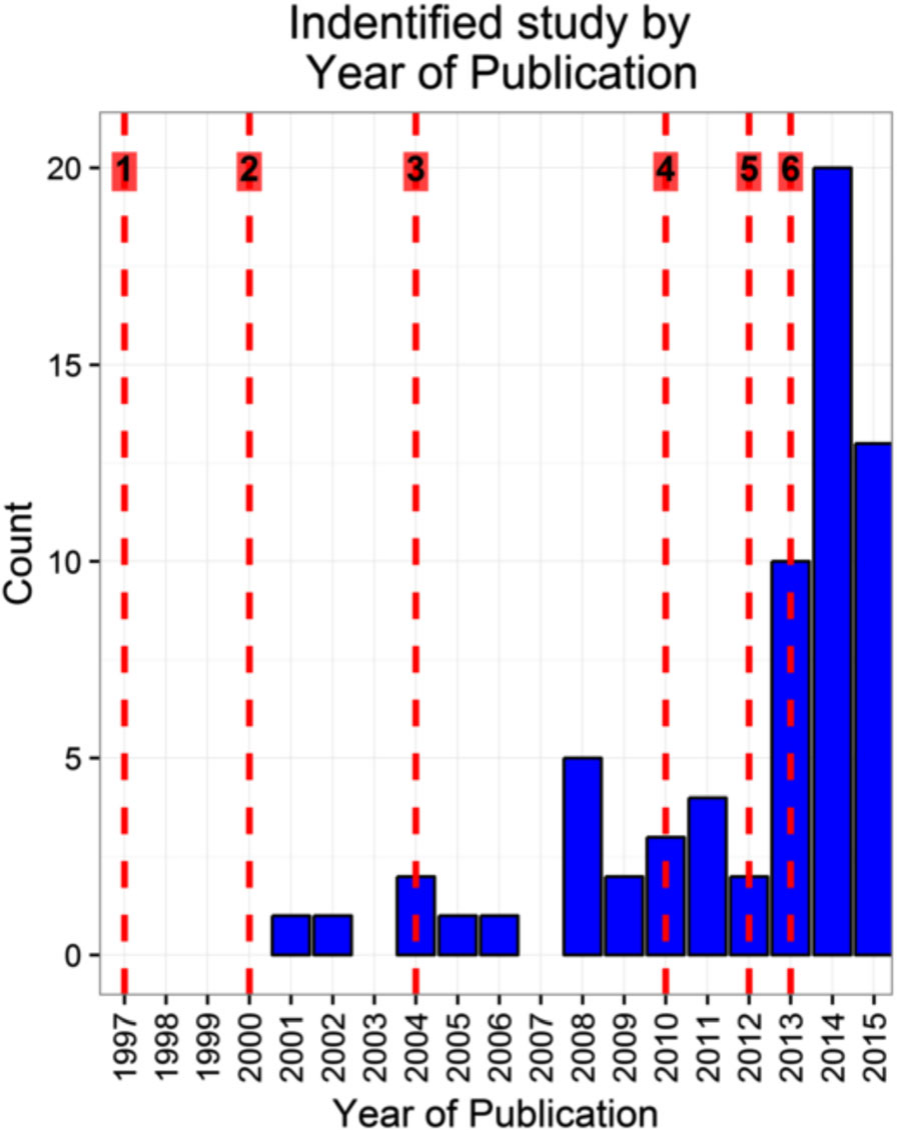
Year of publication of identified studies. Line numbers identify possibly influential publications (see main text)

The distribution of publication year of the studies was skewed towards more recent dates with median publication year 2014 (interquartile range (IQ) 2011–2014, range 2001–2015). Figure 2 indicates an overall trend (with variation between years) of increasing numbers of applied joint modelling papers published (although the maximum number published in a year was only 20). On this graph we have included lines numbered 1–6 at times when significant joint modelling papers were published. In 1997 Wulfsohn and Tsiatis published a paper commonly cited as one of the first joint modelling papers [74] (line 1). In 2000, Henderson et al [9] extended this methodology, with discussion of different sharing structures between the sub-models (line 2). In 2004 two papers were published, by Tsiatis and Davidian [1] (a review of joint modelling methodology), and by Guo and Carlin [75] (examples of implementation of joint modelling in current software) (line 3). In 2010 Rizopoulos published a paper detailing the R joint modelling package JM [76] (line 4), and 2012 saw the publication of a joint modelling textbook [77], and papers describing joint modelling options in Stata (Crowther et al [78]) and the joineR package in R (Philipson et al [79]) (line 5). Also Crowther et al published further papers on joint modelling in Stata in 2013 [80, 81] (line 6). In addition to these events the number of joint modelling workshops, talks and related conferences has increased in recent years (see https://www.liverpool.ac.uk/translational-medicine/departmentsandgroups/joine-r/workshops/, http://eur.academia.edu/DimitrisRizopoulos/Talks, http://www2.le.ac.uk/departments/health-sciences/research/biostats/staff-pages/mjc76, accessed 28 Nov 2016). Whilst it is unclear which of these publications or events contributed to increases in use of joint modelling methods, an increase is noticeable in the application of joint modelling after 2012.

#### Full text or abstract

Full articles were obtained for 63 studies (96.9%) [9–21, 23–54, 56–73], with abstracts available for 2 studies (3.1%) [22, 55]. Of the identified studies, some individuals were listed as authors on multiple studies, suggesting that the group of individuals applying joint modelling methods may be limited.

#### Disease area

The disease areas of the studies were wide ranging (Table 1), with the most common including Cancer, HIV/AIDs, transplant data and cognitive decline. This wide range of disease areas demonstrates the applicability of joint modelling methods to a variety of medical fields, however also indicates that currently finding multiple joint modelling studies applied to the same area to pool in an AD-MA could be problematic.

**Table 1:** Characteristics of identified studies

	*N* (%)
Full text or abstract available	
Full text	63 (96.9)
Abstract	2 (3.1)
Disease Area	
Cancer related data	10 (15.4)
HIV/AIDS	9 (13.8)
Patient status after transplants	8 (12.3)
Cognitive decline	7 (10.8)
Glaucoma	4 (6.2)
Renal disease	4 (6.2)
Disability in the elderly	3 (4.6)
Heart related data	3 (4.6)
Schizophrenia	3 (4.6)
Sclerosis	3 (4.6)
Other	11 (16.9)
Journal	
Statistics in Medicine	5 (7.7)
Journal of the Royal Statistical Society. Series C: Applied Statistics	4 (6.2)
Ophthalmology	3 (4.6)
Quality of Life Research	3 (4.6)
Journal of the American Geriatrics Society	2 (3.1)
Journal of the American Statistical Association	2 (3.1)
Journals of Gerontology - Series B Psychological Sciences and Social Sciences	2 (3.1)
Statistical Methods in Medical Research	2 (3.1)
Other (only one study per journal)	45 (64.6)
Reason for joint modelling use*	
To investigate the link between longitudinal and time-to-event outcomes	43 (66.2)
To account for dropout	22 (33.8)
To include longitudinally measured variable in time-to-event model	4 (6.2)
To increase efficiency	3 (4.6)
To reduce bias	2 (3.1)
Easier to interpret	1 (1.5)
To use of all available data	1 (1.5)

*Note for “disease area” and “journal” only one value was recorded per included study giving total *N* = 65, however for “reason for joint modelling use” multiple reasons could be recorded per included study giving total *N* ≥ 65

#### Journal

The studies identified were published in a range of journals, with 8 journals occurring more than once (Table 1), indicating that there may not currently a preferred journal to present joint modelling studies in.

#### Reason for use of joint model

The reasons given for using joint models are summarised in Table 1 (with some studies providing multiple reasons giving a total greater than 65). The two most common reasons for joint model use were to investigate the link between the outcomes, or to account for dropout. Only 4 studies stated that they used a joint model to include a time varying covariate in the time-to-event sub-model.

#### Source of methods used

Of the included studies 18 (27.7%) used study specific modelling methods, whilst the remainder cited methods described in other papers. In total 38 unique papers were cited for methods, with ten cited by more than one study (see Table 2, references included in table). Some papers were software specific (e.g., Rizopoulos 2010 [76] and 2012 [77] are R related, whilst Crowther et al 2013 [81] is Stata related). Others provided methodology and implementation overviews (e.g., Proust-Lima et al [82] and Guo-Carlin [75]).

**Table 2:** Methods used in identified studies

	*N* (%)
Source of joint modelling methods used	
Own methods developed	18 (27.7)
Guo-Carlin 2004 [75]	13 (20.0)
Rizopoulos 2010 JM R package [76]	10 (15.4)
Henderson et al 2000 [9]	7 (10.8)
Tsiatis and Davidian 2004 [1]	7 (10.8)
Rizopoulos 2012 [77]	6 (9.2)
Wulfsohn and Tsiatis 1997 [88]	6 (9.2)
Diggle et al 2008 [89]	3 (4.6)
Crowther et al 2013 [81]	2 (3.1)
Proust-Lima et al 2009 [82]	2 (3.1)
Rizopoulos 2011 [90]	2 (3.1)
Approach	
Frequentist	45 (69.2)
Bayesian	17 (26.2)
Both	1 (1.5)
Unclear	2 (3.1)
Sharing structure	
Fixed and Random Effects	33 (50.8)
Current Value of Fixed and Random Effects	24 (36.9)
Current Slope (first derivative) of Fixed and Random Effects	3 (4.6)
Current Value of Fixed and Random Effects and Current Slope (first derivative) of Fixed and Random Effects	5 (7.7)
Fixed and random effects without covariates	1 (1.5)
Random Effects only	27 (41.5)
Intercept only	5 (7.7)
Random Effects with covariates	7 (10.8)
Random Effects without covariates	9 (13.8)
Random Effects unclear with or without covariates	6 (9.2)
Latent Class	3 (4.6)
Specialist sharing structure	4 (6.2)
Unclear	4 (6.2)

Note for “Approach” only one value was recorded per included study giving total *N* = 65, however for “Source of joint modelling methods used” and “Sharing structure” multiple reasons could be recorded per included study giving total *N* ≥ 65

#### Modelling approach

Of the 65 included studies, 45 (69.2%) took a frequentist approach, 17 (26.2%) took a Bayesian approach, 1 (1.5%) took both (in separate model fits) and in 2 (3.1%) studies it was unclear (these were the two abstracts). The larger proportion of frequentist approaches may be attributable to the larger number of papers and based on frequentist methods. Additionally the main joint modelling textbook [77] deals with frequentist methods.

There were 21 unique model types recorded for the longitudinal sub-model (plus 1 study with unclear type). Linear mixed effects models were most common (35 studies, 53.8%), followed by mixed effect models with splines (6 studies, 9.2%) or mixed models with unspecified structure (5 studies, 7.7%). Other methods used included different mixed models dependent on latent class, non-linear models with or without splines, and models with change points.

The methods used for the time-to-event sub-model varied widely (and were unclear in 4 studies). The Cox proportional hazards (PH) model was most common (8 studies, 12.3%). Other methods included models with parametric baselines, such as a Weibull PH model (5 studies, 7.7%), a PH model with piecewise constant baseline (4 studies, 6.2%), or a spline modelled baseline hazard (2 studies, 3.1%). Parametric models included the Weibull (5 studies, 7.7%) and the exponential (1 study, 1.5%), and 1 study (1.5%) examined both Weibull and exponential models.

#### Sharing structure between longitudinal and time-to-event sub-models

The structures used to link the longitudinal and time-to-event sub-models are listed in Table 2. Some studies fitted multiple joint models, with varying sharing structures, allowing a total of more than 65 recorded sharing structures.

Any sharing structure (also termed association structure) involving a function of both fixed and random effects is designated “Fixed and Random effects” whereas those involving random effects but no fixed effects are termed “Random Effects only”. Fixed and random effects sharing structures (33 studies, 50.8%) model effects of aspects of the overall longitudinal outcome value on the time-to-event outcome. The random effects only sharing structures (27 studies, 41.5%) model the effect of individual deviation from the population mean longitudinal outcome on the time-to-event outcome. A description of fixed and random effects sharing structures is given in Rizopoulos 2012 [77], whilst Henderson et al [4] discuss random effects only sharing structures. Additionally Rizopoulos and Ghosh [83] and Gould [84] discuss a range of association structures.

The fixed and random effect sharing structures can be subdivided further. Current value refers to models inserting the current longitudinal trajectory value into the time-to-event sub-models, and is used when the current overall value of the longitudinal trajectory affects the risk of an event. The current slope or first derivative of the population trajectory could also be inserted into the time-to-event sub-model, in conjunction with the current value or alone, and is used to model the effect of rate of change of the longitudinal variable on the risk of an event. Another fixed and random effects sharing structure identified in one study, inserted the fixed and random effects coefficients of the longitudinal trajectory into the time-to-event sub-model without their related covariates.

Random effects only sharing structures can also be grouped. We define a random effects only sharing structure to contain covariates if it is of format such as *α*(*U*_0_ + *U*_1_ *t*) or *α*_1_ *U*_0_ + *α*_2_ *U*_1_ + *α*_3_(*U*_0_ + *U*_1_ *t*) where the *α* terms are association parameters, the *U* terms are random effects, and *t* represents a covariate such as time. Alternatively, if the structure is similar to *α*(*U*_0_ + *U*_1_), where the random effect *U*_1_ had a covariate *t* in the longitudinal sub-model, we define the random effects only sharing structure to not contain covariates (see Henderson et al [9] for further examples).

The specialist sharing structure group (4 studies (6.2%)) contained less common sharing structures such as associating the time-to-event and longitudinal sub-models through a multivariate distribution. Another option was the latent class structure [3, 82], used in at least one joint model in 3 studies (4.6%). Finally 4 (6.2%) studies, including the 2 abstracts, had unclear sharing structures.

We should note that choice of association structure should be driven by the data itself, and so it is expected to see a range of sharing structures given the range of disease areas of the identified studies.

#### Software

The software and package used in the included studies is listed in Table 3. Packages/methods have been stated in Table 3 even if no identified studies currently used them.

**Table 3:** Software used in joint model fits in included studies

Software	*N* (%)
R [91]	21 (32.3)
R (JM) [76]	15 (23.1)
R (JMBayes) [92]	0 (0)
R (joineR) [79]	1 (1.5)
R (frailtypack) [93]	0 (0)
R (JM and joineR) [76, 79]	1 (1.5)
R (unspecified package)	2 (3.1)
R (own code developed, unclear if available)	2 (3.1)
SAS [94]	13 (20.0)
SAS (PROC NLMIXED)	10 (15.4)
SAS (own code available)	1 (1.5)
SAS (unspecified)	2 (3.1)
JM Macro [95]	0 (0)
JMFit Macro [96]	0 (0)
Stata [97]	5 (7.7)
Stata (stjm) [80]	2 (3.1)
Stata (unspecified)	3 (4.6)
WinBUGS [98]	4 (6.2)
WinBUGS (own code available)	2 (3.1)
WinBUGS (no available code)	1 (1.5)
WinBUGS (unspecified)	1 (1.5)
OpenBUGS (no available code)	1 (1.5)
Fortran	3 (4.6)
Fortran (code available, not study specific)	1 (1.5)
Fortran (own code developed)	1 (1.5)
Fortran (study states code available)	1 (1.5)
NONMEM (unspecified) [99]	2 (3.1)
C++ (own code unclear if available) [100]	1 (1.5)
Mplus (unspecified) [101]	1 (1.5)
More than one software listed/potentially used	4 (6.2)
R (JM) or SAS (unspecified)	1 (1.5)
R or SAS (unspecified)	1 (1.5)
WinBUGS and R (Directed Acyclic Graph provided)	1 (1.5)
WinBUGS and R (own code available)	1 (1.5)
Unclear	10 (15.4)

Note that studies could report multiple joint fits using different software, so total N ≥ 65. For Mclain [43] R code is stated as available in supplementary material, which was missing when accessed. Lawson [37] may have used WinBUGS but without seeking confirmation from the authors this was classed as unclear software. For Fortran see http://www.fortran.com/, accessed 28 Nov 2016)

Software used was not always stated by identified studies, a potential issue for future MA when determining exact modelling structure used. The most mentioned software was R, although SAS and Stata were also common. Some of the software identified requires more coding from users (such as C++ WinBUGs), which might explain the preference for software with specific joint modelling packages.

The current preference in R is for the JM package [76] (implements frequentist joint models that insert the fixed and random effects of the longitudinal sub-model into the time-to-event sub-model), and PROC NLMIXED in SAS (allows fitting of a range of non-linear mixed models). The popularity of JM might be explained by the availability of a textbook with worked examples of joint model implementation using the package [77].

In four studies (6.2%) more than one software was stated, it was unclear which implemented the joint model fit.

### Did studies report sufficient information to contribute to meta-analyses?

For an identified study to contain sufficient information to contribute to a MA, it must report a sample size. For each meta-analysis group (longitudinal, time-to-event, association), the relevant parameter estimates must be reported with a precision estimate (a standard error, or a confidence interval with related significance level). A summary of this information is given in Table 4.

**Table 4:** Summary of information available to contribute to meta-analysis

	Longitudinal MA	Time-to-event MA	Association MA
Coefficients reported (%)	45 (69.2)	46 (70.8)	51 (78.5)
Precision reported (%)	44 (67.7)	45 (69.2)	50 (76.9)
Standard Errors reported (%)	22 (33.8)	23 (35.4)	25 (38.5)
Confidence Intervals (CI) reported (%)	30 (46.2)	32 (49.2)	36 (55.4)
Significance level reported (%)	57 (87.7)	57 (87.7)	57 (87.7)
Sample size reported (%)	64 (98.5)	64 (98.5)	64 (98.5)
MA possible given reported information (%)			
All identified studies (*N* = 65)	44 (67.7)	45 (69.2)	50 (76.9)
Studies using joint models to account for dropout (*N* = 22)	18 (81.8)	14 (63.6)	15 (68.2)
Studies using joint models to include time varying covariate in time-to-event sub-model (*N* = 4)	2 (50.0)	3 (75.0)	3 (75.0)

The sample size was reported in the majority (98.5%) of the identified studies, with median sample size of 514 (IQR 277–1054.5, range 46–3814).

The association parameters were more commonly reported (51 studies (78.5%)) than those of the longitudinal and time-to-event sub-models (45 (69.2%) and 46 (70.8%) respectively). This could be attributable to the high proportion of identified studies that stated that joint models were used to investigate the link between longitudinal and time-to-event outcomes.

The number of studies where a precision measure was available was comparable to the number of studies that reported coefficients, across the three MA categories. Whilst a MA would be possible for each category if the parameter, the standard error and the sample size were reported, if the only available precision estimate was the confidence interval then the significance level was also required. In the studies we identified, the significance level was unclear for 8 (12.3%) studies, was 0.05 for 53 (81.5%) studies, 0.01 for 3 (4.6%) studies, 0.1 for 1 (1.5%) study.

Overall, a MA would be possible for the association parameter in 50 (76.9%) studies, for the longitudinal parameters in 44 (67.7%) studies, and for time-to-event parameters in 45 (69.2%) studies.

Ideally, a study would provide enough information to perform MA in all three separate groups. Sufficient information to allow all three MA to be undertaken was available from 38 (58.5%) of the studies. Only two MA were possible in 6 studies (9.2%), only 1 in 13 studies (20.0%) and in 8 studies (12.3%) there was not sufficient information to complete any MA.

The reasons for joint model use may affect the information stated in the study report. We re-examined the proportions of studies in which the three groups of MA could take place, dependent on the reason for joint model use (Table 4).

For the 22 studies which stated accounting for dropout as a reason for joint model use, we saw a much higher percentage (81.8%) for which MA of longitudinal sub-model parameters was possible, compared to for all studies (67.7%). However percentages of MA possible for the time-to-event coefficients or association parameters were smaller. This could be explained by studies using joint models to account for dropout being mainly interested in the parameters from the longitudinal sub-model.

Only 4 studies stated inclusion of a time varying covariate in a time-to-event model as one of their reasons for using joint models. There was a slight indication that the longitudinal coefficients for studies using joint models to include time varying covariates in time-to-event models are worse reported than for all identified studies, possibly because the longitudinal component of the joint model is of interest as a covariate rather than an outcome in these cases, however more information is needed before this relationship can be fully investigated.

## Discussion

Joint models for longitudinal and time-to-event data are often stated as beneficial compared to separate longitudinal or time-to-event analyses, as they can reduce bias and increase efficiency in model estimation (see Ibrahim et al 2010 [85] for example). Additionally Powney et al 2014 [86] discuss a study where joint models showed a significant difference between treatment groups that was not identified by separate analyses [87]. These benefits of joint models reinforce the suggestion that in certain circumstances MA of joint models may be more appropriate than MA of separate models.

We aimed through our search strategy to identify all studies that implemented joint models to influence future healthcare, and believe that the studies identified are representative of the current literature. However if studies did not state the key search terms used in this review (see Additionnal file S1 for search strategies) in text accessible to the search, the study may not have been identified. For example, from Powney et al [86] we know that the MAGNETIC trial [87] utilised joint models, however this is not mentioned in the abstract. When joint models are not used as part of the primary analyses, their use may be unclear from the abstract or keywords. Therefore we include statement of statistical methods used in text accessible to search engines to our recommendations for future reporting of joint models, stated in Table 5.

**Table 5:** Recommendations for future reporting of joint models

1. Model structure (longitudinal and time-to-event sub-models, and association structure) be clearly stated
2. Estimates of all model coefficients and their precisions be available in main or supplementary material
3. Software (and packages) used to fit models be stated, or code available on request from author
4. Statistical methods should be mentioned in text accessible to search engine to aid identification of papers in meta-analyses

With the increasing use of joint models in the literature, ensuring they are well reported is vital so that the analyses can be interpreted fully and that the published data can be used in future evidence synthesis. We have identified that for the scenario of AD-MA of the results of joint models published in the literature, it was possible to perform MA from a high proportion but not all studies. We would recommend for future practice that regardless of the reason for joint model use, full model covariates with precision estimates be reported either in the study report or supplementary materials (Table 5) not only to aid interpretation within the study itself, but to ensure that future MA would be possible.

Additionally it is important that model structure (both sub-models and the sharing structures that link them) are clearly reported (Table 5). However in some cases model formulae were not reported, a particular issue identified for association parameters. We have noted the multiple association structures available to researchers. Due to the different interpretation of each, it may not make sense to pool association parameters from radically different sharing structures. Without clear statement of the model structure it may be difficult to conduct a future MA. It would be beneficial if studies applying joint models included statement of the model structure as standard to give clarity to the methods used.

Also, it is important especially in joint modelling to state the software and packages or functions used, as the software used could indicate the model structure, as well as methods used to obtain the parameter estimations (Table 5).

Consistently, the proportion of studies where association parameter MA was possible was higher than the proportion where MA was possible for longitudinal or time-to-event parameters. The association information may have been more commonly reported than the longitudinal or time-to-event information because the association parameters in shared random effect models quantify the link between the sub-models. It can be expected therefore that studies aiming to quantify the link between the sub-models report the association information more prominently than other model parameters.

## Conclusion

Overall, this investigation has highlighted the need to fully report the coefficients and precision estimates in studies applying joint models to datasets. Whilst this review identified a limited number of studies that fulfilled our criteria, the range of disease areas covered by the studies was large with applications using a wide range of sub-models and association structures being published in an assortment of journals by a range of authors. It may be some years before there are sufficient studies published in one area to conduct a meta-analysis of joint models. Nevertheless our review has demonstrated that the use of joint models is increasing every year and the availability of software to fit a range of flexible models is likely to facilitate their application even further.

In the future the current standard of joint modelling should be maintained and improved upon, following the recommendations given in this paper, in order that information from published studies can be used for the purpose of MA.

## Additional files

Additional file 1: This file includes the search strategies used in this review to search Medline, Pubmed and Scopus. (DOCX 13 kb)
Additional file 2: This file includes a blank example of the data collection form used to record information from the studies identified by this review. (DOCX 14 kb)
