# Correction to: joint models for longitudinal and time-to-event data: a review of reporting quality with a view to meta-analysis

**DOI:** 10.1186/s12874-018-0489-7

**Published:** 2018-04-04

**Authors:** Maria Sudell, Ruwanthi Kolamunnage-Dona, Catrin Tudur-Smith

**Affiliations:** Department of Biostatistics, Block F Waterhouse Building, University of Liverpool, 1-5 Brownlow Street, Liverpool, L69 3GL UK

## Correction

Following publication of the original article [1] the authors reported that reference 15 (Cella et al.) had been incorrectly replaced with a duplicate of Brombin et al. during publication. Reference 15 should read as follows:

Cella, D., et al., *Survival-adjusted health-related quality of life (HRQL) among patients with metastatic breast cancer receiving paclitaxel plus bevacizumab* versus *paclitaxel alone: Results from Eastern Cooperative Oncology Group Study 2100 (E2100).* Breast Cancer Research and Treatment, 2011. 130(3): p. 855–861.
