# Supplementary material for: Joint models for longitudinal and time-to-event data: a review of reporting quality with a view to meta-analysis
Source: BMC Med Res Methodol. 2016 Dec 5;16:168. doi: 10.1186/s12874-016-0272-6 (PMC5139124; doi:10.1186/s12874-016-0272-6)
Supplement: Additional file 1: — This file includes the search strategies used in this review to search Medline, Pubmed and Scopus. (DOCX 13 kb) [file 12874_2016_272_MOESM1_ESM.docx]

Supplemental material for Review of reporting of joint models

Database – Scopus

( TITLE-ABS-KEY ( joint W/3 model* ) ) AND ( ( TITLE-ABS-KEY ( longitudinal W/4 survival ) ) OR ( TITLE-ABS-KEY ( longitudinal W/4 "time-to-event" ) ) OR ( TITLE-ABS-KEY ( longitudinal W/4 ( time W/3 event ) ) ) OR ( TITLE-ABS-KEY ( "repeat* measure*" W/4 survival ) ) OR ( TITLE-ABS-KEY ( "repeat* measure*" W/4 "time-to-event" ) ) OR ( TITLE-ABS-KEY ( "repeat* measure*" W/4 ( time W/3 event ) ) ) )

Database – PubMed

(joint model*) AND (((((((((longitudinal and survival)) OR (longitudinal and "time-to-event")) OR (longitudinal and "time to event")) OR (longitudinal and "event time")) OR ((repeat* measure*) and survival)) OR ((repeat* measure*) and "time-to-event")) OR ((repeat* measure*) and "time to event")) OR ((repeat* measure*) and "event time"))

Database: Ovid MEDLINE(R) and Ovid OLDMEDLINE(R) <1946 to Present with Daily Update>

Search Strategy:

--------------------------------------------------------------------------------

1 (joint adj3 model*).mp. [mp=title, abstract, original title, name of substance word, subject heading word, keyword heading word, protocol supplementary concept word, rare disease supplementary concept word, unique identifier] (2329)

2 (longitudinal adj4 survival).mp. [mp=title, abstract, original title, name of substance word, subject heading word, keyword heading word, protocol supplementary concept word, rare disease supplementary concept word, unique identifier] (283)

3 (longitudinal adj4 time-to-event).mp. [mp=title, abstract, original title, name of substance word, subject heading word, keyword heading word, protocol supplementary concept word, rare disease supplementary concept word, unique identifier] (39)

4 (longitudinal adj4 (time adj3 event)).mp. [mp=title, abstract, original title, name of substance word, subject heading word, keyword heading word, protocol supplementary concept word, rare disease supplementary concept word, unique identifier] (54)

5 ("repeat* measure*" adj4 survival).mp. [mp=title, abstract, original title, name of substance word, subject heading word, keyword heading word, protocol supplementary concept word, rare disease supplementary concept word, unique identifier] (37)

6 ("repeat* measure*" adj4 time-to-event).mp. [mp=title, abstract, original title, name of substance word, subject heading word, keyword heading word, protocol supplementary concept word, rare disease supplementary concept word, unique identifier] (10)

7 ("repeat* measure*" adj4 (time adj3 event)).mp. [mp=title, abstract, original title, name of substance word, subject heading word, keyword heading word, protocol supplementary concept word, rare disease supplementary concept word, unique identifier] (15)

8 2 or 3 or 4 or 5 or 6 or 7 (367)

9 1 and 8 (102)

***************************
