# Supplementary material for: Joint models for longitudinal and time-to-event data: a review of reporting quality with a view to meta-analysis
Source: BMC Med Res Methodol. 2016 Dec 5;16:168. doi: 10.1186/s12874-016-0272-6 (PMC5139124; doi:10.1186/s12874-016-0272-6)
Supplement: Additional file 2: — This file includes a blank example of the data collection form used to record information from the studies identified by this review. (DOCX 14 kb) [file 12874_2016_272_MOESM2_ESM.docx]

Data Collection form Review of reporting of joint models in the literature

| **General information** |  |
| --- | --- |
| Title |  |
| Authors |  |
| Journal |  |
| Year |  |
| Reference |  |
| Sharing structure between sub-models (longitudinal current value, slope, current and slope, random effects proportional, random effects separate, latent classes) |  |
| Joint modelling method |  |
| Bayesian or frequentist |  |
| Details of fitting methods |  |
| Software |  |
| Population |  |
| Disease Area |  |
| Time-to-event variable(s) |  |
| Longitudinal variable(s) |  |
| Length of follow-up for longitudinal |  |
| Number of longitudinal measurements |  |
| Main reason for joint model use |  |
| **Main model** |  |
| Sample size reported |  |
| Adjusted or unadjusted |  |
| Longitudinal treatment effect reported |  |
| Longitudinal treatment effect standard error/variance reported |  |
| Longitudinal treatment effect confidence interval reported |  |
| Time-to-event treatment effect reported |  |
| Time-to-event treatment effect standard error/variance reported |  |
| Time-to-event treatment effect confidence interval reported |  |
| Association estimate reported |  |
| Association estimate standard error/variance reported |  |
| Association estimate confidence interval reported |  |
